# Structured Pharmacist-Led Vaccine Recommendation and Coadministration in Community Pharmacies: Real-World Evidence from Poland

**DOI:** 10.3390/vaccines14090823

**Published:** 2026-09-19

**Authors:** Ewa Kuczwalska, Anna Maria Dworakowska, Magdalena Skarżyńska, Magdalena Bujalska-Zadrożny

**Affiliations:** 1 Department of Pharmacotherapy and Pharmaceutical Care, Faculty of Pharmacy, Medical University of Warsaw, ul. Banacha 1, 02-097 Warsaw, Poland; 2 Clinical Trials Department, Center of Hearing and Speech, ul. Mokra 7, 05-830 Kajetany, Poland; 3 Institute of Sensory Organs, ul. Mokra 1, 05-830 Kajetany, Poland

**Keywords:** pharmacist-led vaccination, vaccine coadministration, adult immunization, RSV, influenza, COVID-19, community pharmacy, implementation research, real-world evidence

## Abstract

**Background and Objectives:** Community pharmacies in Poland administer an expanding range of adult vaccines, including influenza, COVID-19, respiratory syncytial virus (RSV), herpes zoster and pneumococcal vaccines. While pharmacist vaccination services are increasingly available, evidence on structured approaches supporting vaccine coadministration remains limited. This study assessed whether a structured pharmacist-led recommendation and coadministration protocol was associated with greater uptake of multiple vaccines and same-day coadministration in routine community pharmacy practice. **Methods:** This non-randomized real-world study used anonymized vaccination records from three community pharmacies in northern Poland between 1 August 2025 and 31 March 2026. One pharmacy implemented a multicomponent protocol combining proactive identification of eligible patients, presumptive recommendation and same-day coadministration, while two comparator pharmacies operated under routine practice. Outcomes included the mean number of vaccine types per patient, the proportion of patients receiving at least two of five analyzed vaccines, same-day coadministration among multi-vaccine patients, and the RSV-to-(influenza + COVID-19) vaccination ratio. A Poisson regression model with robust standard errors was used to assess relative RSV uptake. As the dataset comprised only patients who received at least one vaccination, these findings describe vaccination patterns among presenting, already-vaccinated patients rather than uptake relative to the total eligible or registered pharmacy population, for which denominators were not available. **Results:** Overall, 3248 patients contributed 5505 unique patient-vaccine events. The intervention pharmacy achieved a higher mean number of vaccine types per patient than comparator pharmacies (2.08 vs. approximately 1.54). The proportion of patients receiving at least two vaccine types was 73.6% (685/931) in the intervention pharmacy compared with 42.7% (990/2317) in comparator pharmacies. Among multi-vaccine patients, same-day coadministration occurred in 92.7% (635/685) of intervention-site patients versus 73.1% (724/990) in comparator pharmacies. Triple-vaccine coadministration visits were observed exclusively in the intervention pharmacy. RSV uptake was higher in the intervention pharmacy (incidence rate ratio 2.17; 95% CI 1.67–2.84; *p* < 0.001). An age- and sex-adjusted analysis of multi-vaccine uptake yielded a materially unchanged association (adjusted OR 4.42; 95% CI 3.71–5.28) relative to the unadjusted estimate (OR 3.73; 95% CI 3.16–4.41). The exploratory temporal-interaction analysis was not statistically significant when all months were included (interaction IRR 0.84; 95% CI 0.69–1.02; *p* = 0.085), whereas a sensitivity analysis excluding March 2026 suggested a narrowing relative RSV-rate advantage over time (interaction IRR 0.79; 95% CI 0.70–0.89; *p* < 0.001). **Conclusions:** A structured pharmacist-led recommendation and coadministration protocol was associated with substantially greater uptake of multiple vaccines among vaccinated patients, more frequent same-day coadministration and higher RSV uptake compared with routine practice. These findings suggest that workflow organization, proactive patient identification and systematic recommendation strategies may improve the efficiency of adult vaccination delivery in community pharmacies. Larger multicenter studies are warranted to confirm these findings and evaluate individual protocol components.

## 1. Introduction

Respiratory syncytial virus (RSV), seasonal influenza and COVID-19 remain major causes of morbidity, hospitalization and mortality among older adults and individuals with chronic conditions such as cardiovascular disease, chronic lung disease, diabetes and immunosuppression [1,2,3]. Effective vaccines targeting these infections, as well as vaccines against herpes zoster and pneumococcal disease, are currently available and increasingly delivered in community pharmacy settings in many countries [4,5,6,7], including Poland [8].

In Poland, the scope of pharmacy-based vaccination services has expanded substantially since 2021. The main factor that triggered legislative changes in this area was the COVID-19 pandemic [9]. Community pharmacies now administer a broad portfolio of adult vaccines, including those against influenza, COVID-19, RSV, herpes zoster and pneumococcal vaccines [8,10]. Reimbursement policies support uptake, with full reimbursement of COVID-19 vaccination for all adults and full reimbursement of RSV and influenza vaccination for individuals aged ≥65 years, while adults aged 18–64 years may access these vaccines with partial co-payment [8].

Despite progress, vaccination coverage among adults still remains suboptimal [11,12]. International evidence indicates that simply enabling pharmacists to vaccinate and ensuring reimbursement is insufficient to maximize uptake [6]. Structural and organizational barriers, including fragmented workflows, lack of standardized protocols, and limited use of systematic patient engagement strategies, continue to result in missed opportunities for vaccination [4,5,13,14,15]. The cost of vaccination may also represent an important barrier to vaccine uptake [16,17]. For example, in Poland the price of the RSV vaccine for individuals under 65 years of age who are not eligible for full reimbursement may be perceived as high and may discourage some patients from being vaccinated [18].

Coadministration of vaccines during a single visit is a well-established strategy to reduce missed opportunities and improve vaccination coverage, particularly in populations at increased risk [5,19,20,21,22]. Clinical and real-world data demonstrate that coadministration of influenza and COVID-19 vaccines maintains immunogenicity and has an acceptable safety profile, while emerging evidence suggests that RSV vaccines can be safely integrated into coadministration strategies in adults [21,23]. This safety evidence base spans both younger and older adults and is discussed in greater detail, together with additional age-stratified citations, in Section 4. However, in routine practice, coadministration is often underutilized and tends to depend on opportunistic rather than systematic decision-making [24].

Importantly, growing evidence from behavioral and implementation science suggests that vaccination uptake is strongly influenced not only by availability but also by how vaccination is recommended [25]. In particular, a clear, proactive and personalized “strong recommendation”, combined with default or opt-out framing, can substantially increase acceptance compared with passive or optional offers. In pharmacy practice, such approaches may be operationalized through structured protocols that ensure each eligible patient is systematically assessed and offered a complete vaccination package during a single encounter.

Previous studies have evaluated pharmacy-based immunization record review, personalized vaccine recommendations, and vaccine coadministration in community pharmacies [14]; however, evidence remains limited on integrated pharmacy-wide protocols that combine proactive patient identification, presumptive recommendation, and systematic same-day coadministration using real-world vaccination records.

The aim of this study was to evaluate the association of a structured pharmacist-led recommendation and coadministration protocol with adult vaccination patterns in real-world community pharmacy practice, with a focus on receipt of multiple vaccine types and same-day coadministration.

## 2. Materials and Methods

### 2.1. Study Design and Setting

This retrospective, non-randomized real-world study used anonymized vaccination records from three community pharmacies of the same owner and covered the period from 1 August 2025 to 31 March 2026, although Kobylnica’s routine reporting in the national system began in September 2025 (Section 2.5), which is why September 2025 rather than August 2025 was used as the statistical reference month. The three pharmacies were located in neighbouring towns in the Pomeranian Voivodeship of northern Poland: Lębork (approximately 35,000 inhabitants), Słupsk (approximately 90,000 inhabitants) and Kobylnica, a suburban municipality directly adjacent to Słupsk. The distance between study sites ranged from approximately 15 to 55 km.

One pharmacy in Lębork implemented a structured pharmacist-led coadministration protocol, while two comparator pharmacies in Słupsk and Kobylnica operated under routine practice without a formal protocol.

The intervention pharmacy (Lębork) was selected pragmatically rather than randomly. In the owner’s and vaccinating pharmacist’s a priori judgement, Lębork had the least favourable conditions among the three sites for spontaneous, patient-initiated vaccination demand: it is the smallest of the three towns, and the pharmacy’s location does not benefit from adjacent referral flow, in contrast to Słupsk (located opposite a primary-care clinic that refers patients for vaccination) and Kobylnica (located inside a shopping centre with substantial pedestrian footfall). The structured protocol was therefore implemented at the site judged, before the study, least likely to generate high vaccination volume without active staff intervention, rather than at a site already expected to perform well. This site assessment was qualitative and was not based on documented pre-implementation vaccination data, so it cannot be verified retrospectively, and residual confounding by unmeasured site-level factors cannot be excluded (Section 4). Systematically recorded pre-implementation (pre-August 2025) vaccination counts comparable in structure to the study dataset were not available for any of the three pharmacies. No objective pre-intervention vaccination metrics were available for any site; therefore, site selection was not informed by historical vaccination performance. This is compounded by a substantial change during 2025 to the legal and reimbursement framework governing pharmacy-based vaccination in Poland (Section 1): community pharmacists gained the authority to issue publicly reimbursed prescriptions for vaccines on the national reimbursement list from 14 February 2025 [26], and the RSV vaccine products relevant to this study were added to that list only progressively—Abrysvo (Pfizer Europe MA EEIG, Brussels, Belgium) from 1 April 2025 [27] and Arexvy (GlaxoSmithKline Biologicals S.A., Rixensart, Belgium) from 1 October 2025 [28]—partway through and largely after, respectively, any period that might otherwise have served as a historical baseline. Consequently, even where earlier vaccination counts exist, they were generated under a materially different legal, reimbursement and product-availability environment for most of the analyzed vaccine types and would not be directly comparable to study-period data; this regulatory timeline also explains the choice of Abrysvo, rather than Arexvy, as the RSV product used throughout the study period (Section 2.2). Together, these factors preclude a formal pre/post baseline comparison and are discussed further as a limitation (Section 4).

All three sites used the same national reporting system (Gabinet.gov.pl/Centrum e-Zdrowie), vaccine portfolio and reimbursement rules, and were broadly comparable operationally, with similar opening hours and vaccination days and one vaccinating pharmacist per site [29,30].

The study is reported in accordance with the STROBE checklist for observational studies [31]; a completed checklist is provided as Supplementary Material (Table S4).

### 2.2. Participants and Data Source

The analysis included all recorded vaccinations against COVID-19, influenza, RSV, herpes zoster and pneumococcal disease administered to adults (aged ≥ 18 years) at the three pharmacies during the study period. For each vaccination event, the dataset contained the pharmacy identifier, vaccination date, settlement month, anonymized patient identifier, vaccine type, age and sex. Data were obtained from the national vaccination reporting system (Gabinet.gov.pl, Centrum e-Zdrowie) and are subject to a data-use agreement.

The primary unit of analysis was the patient. A patient–vaccine event was defined as one unique combination of pharmacy, anonymized patient identifier and vaccine type during the study period; a second dose of the same vaccine type for the same patient (e.g., the second dose of the two-dose herpes zoster series) was therefore counted once within that vaccine type, consistent with the outcome definition in Section 2.4.

Same-day coadministration was defined as two or more different vaccine types recorded for the same anonymized patient identifier in the same pharmacy on the same calendar date; because individual visit identifiers were not available in the reporting system, calendar date was used as the operational proxy for a single vaccination encounter. Patient identifiers were anonymized separately within each pharmacy (site-specific prefixes) and could not be linked across sites; vaccination records could therefore not be attributed to the same individual across different pharmacies, and cross-pharmacy coadministration was not assessed.

Because the dataset was restricted to patients who received at least one of the analyzed vaccines, all uptake-related comparisons in this study are relative comparisons among vaccinated patients across pharmacies, not estimates of uptake against the total eligible or registered catchment population, which was not available from the data source.

Vaccine products used during the study period were identified from the vaccine-name field of the source records. A single COVID-19 vaccine product (Spikevax LP.8.1, Moderna BIOTECH SPAIN, S.L., Madrid, Spain) was administered at all three pharmacies throughout the study period. Influenza vaccination used three quadrivalent products—Influvac Tetra (Viatris Healthcare Limited, Dublin, Ireland), VaxigripTetra (Sanofi Winthrop Industrie, Gentilly, France) and, less frequently, the high-dose product Efluelda Tetra (Sanofi Winthrop Industrie, Gentilly, France) (used almost exclusively in older adults)—consistent with the WHO-recommended composition for the 2025–2026 northern hemisphere season (an A/Wisconsin/67/2022 (H1N1)pdm09-like virus, an A/District of Columbia/27/2023 (H3N2)-like virus, a B/Austria/1359417/2021 (Victoria lineage)-like virus, and, in quadrivalent vaccines, a B/Phuket/3073/2013 (Yamagata lineage)-like virus) [32]. RSV vaccination used a single product, Abrysvo (Pfizer Europe MA EEIG, Brussels, Belgium, bivalent RSVpreF), at all three sites; its EU marketing authorization was extended in April 2025 from adults ≥ 60 years to all adults ≥ 18 years [33], although Polish public reimbursement during the study period remained restricted to patients ≥ 60 years (50% co-payment for ages 60–64, fully reimbursed from age 65) [34], consistent with the age-related reimbursement barrier discussed in Section 1. Abrysvo, rather than the alternative RSV product Arexvy (GlaxoSmithKline Biologicals S.A., Rixensart, Belgium), was used throughout the study period because Abrysvo was added to the national vaccine reimbursement list on 1 April 2025, whereas Arexvy was not added until 1 October 2025, partway through the observation period (Section 2.1); this timing, rather than clinical preference, accounts for Abrysvo being the only RSV product administered at any of the three pharmacies. Herpes zoster vaccination used a single product, Shingrix (GlaxoSmithKline Biologicals S.A., Rixensart, Belgium, recombinant zoster vaccine), and pneumococcal vaccination used a single product, Prevenar 13 (Pfizer Europe MA EEIG, Brussels, Belgium, 13-valent pneumococcal conjugate vaccine), at all three sites; both are included on the national reimbursement list, but, unlike the age-based eligibility criteria for the COVID-19, influenza and RSV vaccines above, reimbursement eligibility for these two vaccines depends on documented medical indications (e.g., specific chronic-disease or immunocompromising conditions) rather than age alone. Because Polish community pharmacists do not have routine access to patients’ complete medical records, they cannot independently verify these indications, and in practice can generally only vaccinate against herpes zoster or pneumococcal disease when the patient already holds a prescription issued by a physician; this operational constraint, rather than a data-recording gap, is the most likely explanation for the comparatively low uptake of these two vaccines observed at all three pharmacies and is discussed further as a limitation in Section 4.

### 2.3. Intervention and Comparator

The intervention pharmacy (Lębork) implemented a multicomponent, team-based recommendation and coadministration protocol that differed from routine practice [35] in two principal respects. First, rather than vaccinating only patients who requested a specific vaccine, the workflow was designed so that the whole pharmacy team would proactively identify potentially eligible adults during routine pharmacy encounters—including visits for prescription dispensing or medication collection—by screening for age and other visible eligibility criteria. Second, eligible patients were intended to receive a structured, presumptive (“strong”) recommendation for all due vaccines, supported by a brief vaccination-history review and a same-day coadministration offer whenever clinically appropriate. The protocol comprised seven sequential steps—patient identification, eligibility assessment, vaccination history review, strong recommendation, offer of same-day coadministration (with scheduling of a subsequent appointment where same-day vaccination was declined or not clinically possible), administration of all eligible vaccines during the same visit, and documentation in Gabinet.gov.pl—with defined responsibilities distributed across the pharmacy team (Supplementary Table S5; Figure S1). The comparator pharmacies (Słupsk, Kobylnica) operated under routine, non-standardized practice in which coadministration was possible but depended on the pharmacist’s clinical judgement and on patient-initiated requests, without systematic proactive identification of eligible patients. Because full medical records were not available to pharmacists at any site, eligibility for reimbursement-dependent vaccines was inferred from age, available documentation and information visible in the e-health system.

The protocol was implemented as part of routine workflow from 1 August 2025, the start of the observation window; adherence to the individual protocol steps was not formally measured.

All vaccines administered during the study period were used within their approved marketing authorization and in accordance with national immunization recommendations. Coadministration of influenza, COVID-19, RSV, herpes zoster and pneumococcal vaccines is explicitly supported by Polish expert guidance on the coadministration of respiratory vaccines in adults [19] and by international clinical guidance, including that of the U.S. Centers for Disease Control and Prevention [20] and other bodies (age-stratified safety evidence is reviewed in Section 4). Each vaccination—whether given alone or coadministered—was a routine clinical act, preceded by standard pre-vaccination screening and written informed consent obtained directly by the vaccinating pharmacist, as required for every vaccination administered in community pharmacies in Poland irrespective of this study. No vaccine was administered outside its licensed indication; no experimental product, dose or schedule was used; and no random allocation, blinding or protocol-driven modification of clinical decision-making occurred. The structured protocol at the intervention pharmacy changed only how eligible patients were identified and how vaccination was recommended and organized among staff (see steps above); it did not alter the individualized clinical decision to vaccinate, which remained with the vaccinating pharmacist and patient at each encounter. This study therefore constitutes a retrospective secondary analysis of routinely collected, anonymized clinical vaccination data rather than a clinical trial, consistent with the Bioethics Committee opinion obtained for this analysis (Institutional Review Board Statement).

### 2.4. Outcomes

The main outcomes were the number of patients receiving at least one analyzed vaccine, the number of unique patient–vaccine events, the mean number of vaccine types per patient, and the proportion of patients receiving at least two analyzed vaccines. Among patients receiving more than one analyzed vaccine, we calculated the proportion with at least one same-day coadministration event and the proportion who received additional vaccines only on separate days. At the visit level, we identified the most frequent same-day vaccine combinations.

A secondary outcome, specified a priori, was the RSV-to-(influenza + COVID-19) vaccination ratio, used as a proxy for the relative uptake of RSV vaccination. Influenza and COVID-19 vaccines were selected as the denominator because they were already delivered in high volumes in all three pharmacies and therefore approximate the overall volume of respiratory vaccination encounters against which RSV uptake could be compared. As an additional exploratory analysis, we fitted an age- and sex-adjusted logistic regression model for the outcome of receiving at least two of the five analyzed vaccines, to assess whether the association between pharmacy group and multi-vaccine uptake was materially altered by the modest between-site difference in patient age.

### 2.5. Statistical Analysis

We performed descriptive analyses to characterize vaccination uptake and coadministration patterns across the three pharmacies. To assess the use of vaccination opportunities, we fitted a Poisson generalized linear model with a log link for the monthly number of RSV vaccinations, with the log of the number of influenza and COVID-19 vaccinations as an offset term and with pharmacy group (intervention vs. comparator) and calendar month as covariates. This rate-based specification estimates the RSV vaccination rate per respiratory vaccination encounter while adjusting for site and seasonal period, and was preferred over simple proportions or logistic regression because the data were aggregated as counts over pharmacy–months with denominators that varied substantially between sites and months. September 2025 was used as the reference month (the first calendar month for which all three pharmacies reported data).

Inspection of model fit indicated overdispersion (Pearson χ^2^/degrees of freedom ≈ 5.5; deviance/degrees of freedom ≈ 5.5). Robust (sandwich) standard errors were therefore used for inference, and the inferential results should be interpreted as exploratory given the small number of site–months available (n = 23). Results are reported as incidence rate ratios (IRRs) with 95% confidence intervals; *p* < 0.05 was considered statistically significant.

To examine whether temporal trends differed between the intervention and comparator pharmacies, we additionally performed an exploratory temporal-interaction analysis by adding a Lębork × calendar-month (linear) interaction term to the model, which tests for a differential linear trend in the RSV rate between sites. Because the March 2026 offset (influenza + COVID-19 vaccinations) fell to 1–3 per site and yielded an unstable endpoint, a sensitivity analysis excluding March 2026 is also reported. No formal pre/post parallel-trends test was performed, because the protocol was implemented at the start of the observation window and a distinct, extended pre-period was not available. No formal sample size calculation was performed; the study was descriptive and used the complete routinely collected data for the study period.

As a complementary patient-level analysis, we fitted a logistic regression model for the binary outcome of receiving at least two of the five analyzed vaccines, with pharmacy group, patient age (years) and sex as covariates, to assess whether the between-site association was confounded by the modest age difference between the intervention and comparator pharmacies. Results are reported as odds ratios (ORs) with 95% confidence intervals.

## 3. Results

A total of 3248 patients received at least one of the five analyzed vaccines during the study period across the three participating pharmacies, contributing 5505 unique patient-vaccine events. The study population included 931 patients in the intervention pharmacy (Lębork), 1424 in Słupsk and 893 in Kobylnica. Baseline demographic characteristics are presented in Table 1.

The intervention pharmacy (Lębork) recorded 1935 unique patient–vaccine events among 931 vaccinated patients, corresponding to a mean of 2.08 vaccine types per patient, compared with 1.53 and 1.55 in Słupsk and Kobylnica, respectively. Thus, patients vaccinated in the intervention pharmacy were associated with approximately 35% more vaccine types per patient than those vaccinated in comparator pharmacies. Vaccination activity and coadministration outcomes are presented in Table 2.

Overall, 1675 patients (51.6%) received at least two of the analyzed vaccines. The proportion of patients receiving ≥ 2 vaccine types was substantially higher in the intervention pharmacy than in the comparator pharmacies (73.6% [685/931] vs. 42.7% [990/2317]), corresponding to a relative likelihood of 1.72 and an absolute difference of 30.9 percentage points.

Because mean patient age differed modestly between the intervention pharmacy and the comparator pharmacies, we fitted an age- and sex-adjusted logistic regression model for the outcome of receiving at least two of the five analyzed vaccines. The unadjusted odds ratio for the intervention pharmacy versus comparator pharmacies was 3.73 (95% CI 3.16–4.41); after adjustment for age and sex, the odds ratio was 4.42 (95% CI 3.71–5.28), materially unchanged from—in fact slightly higher than—the unadjusted estimate. Older age was independently associated with modestly higher odds of receiving ≥ 2 vaccine types (OR 1.042 per year, *p* < 0.001), whereas sex was not significantly associated with the outcome (male vs. female OR 1.04, *p* = 0.63). These results indicate that the between-site age difference does not account for the substantially higher multi-vaccine uptake observed at the intervention pharmacy.

Among multi-vaccine patients, 1359 (81.1%) experienced at least one same-day coadministration event. The proportion of multi-vaccine patients with same-day coadministration was highest in Lębork (635/685; 92.7%), compared with 67.2% (407/606) in Słupsk and 82.6% (317/384) in Kobylnica. Conversely, administration of additional vaccines exclusively on separate days occurred in only 7.3% of multi-vaccine patients in Lębork, compared with 26.9% in the pooled comparator pharmacies, corresponding to an observed 73% relative difference in missed coadministration opportunities. The full breakdown of same-day vaccine combinations by pharmacy is presented in Table 3.

RSV vaccination was administered to 519 patients in Lębork, 317 in Słupsk and 201 in Kobylnica. Influenza and COVID-19 vaccines were the most frequently administered vaccines in all three pharmacies, whereas herpes zoster and pneumococcal vaccines were uncommon. The most frequent same-day combination in Lębork was COVID-19 + influenza + RSV, followed by COVID-19 + influenza and influenza + RSV. Notably, three-vaccine coadministration visits were observed exclusively in the intervention pharmacy, while all same-day coadministration visits in comparator pharmacies involved only two vaccines.

**Figure 1:**
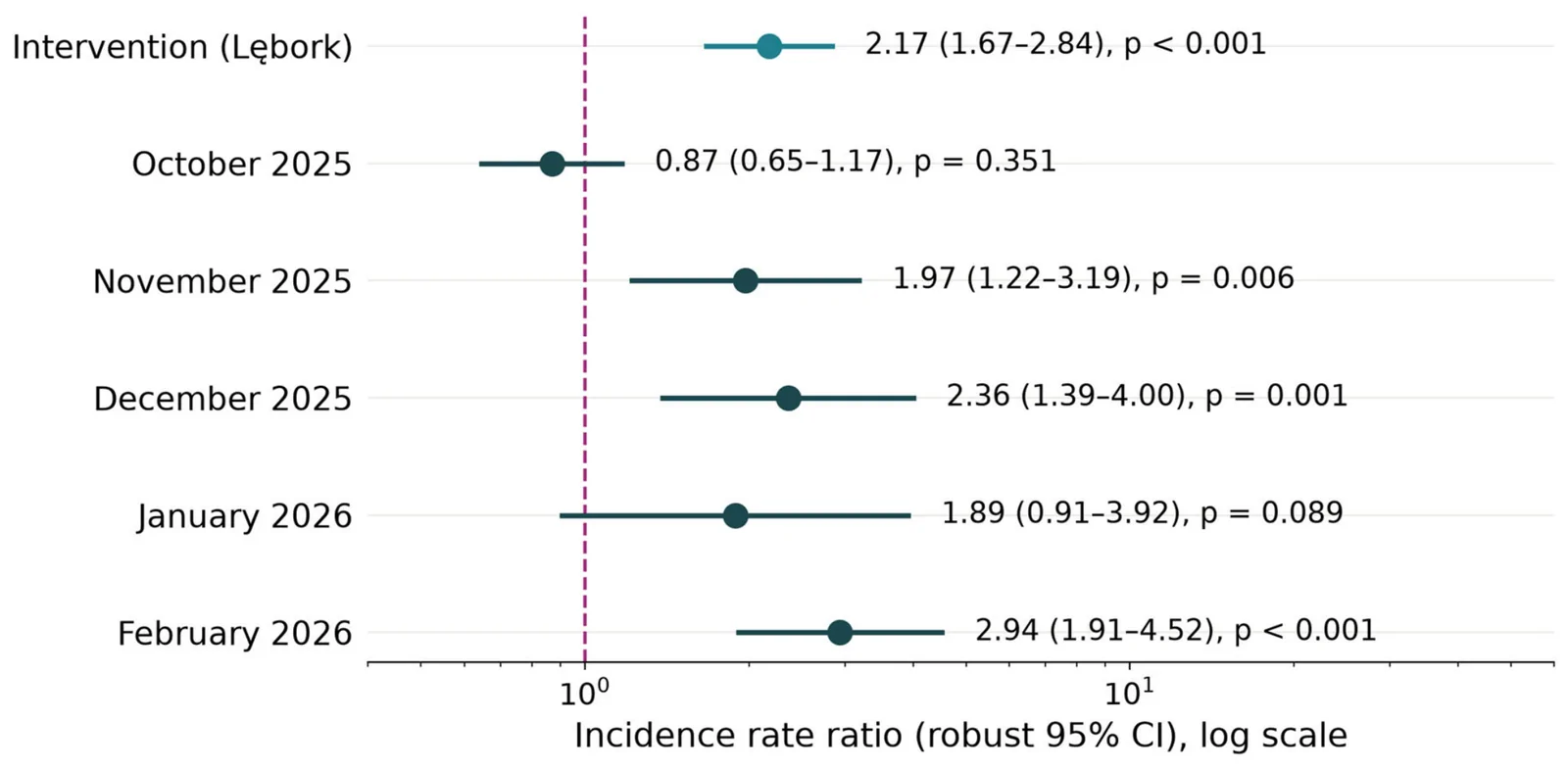
Incidence rate ratios (IRRs) with robust 95% confidence intervals from the Poisson regression model for the RSV vaccination rate (offset: influenza + COVID-19 vaccinations; reference: comparator pharmacies, September 2025). The dashed vertical line indicates IRR = 1 (no difference). March 2026 is not displayed because the offset fell to 1–3 per site, yielding an unstable estimate; this estimate is retained and reported in Supplementary Table S1.

In the Poisson regression model, the intervention pharmacy had a higher RSV vaccination rate per respiratory vaccination encounter than the comparator pharmacies (IRR 2.17; 95% CI 1.67–2.84; *p* < 0.001) after adjustment for calendar month (Figure 1; Supplementary Table S1). Given the small number of underlying site-month observations (n = 23) and the overdispersion noted in Section 2.5, this estimate should be interpreted as exploratory and hypothesis-generating rather than as a precise effect estimate, and larger, multi-pharmacy datasets are needed to refine it.

**Figure 2:**
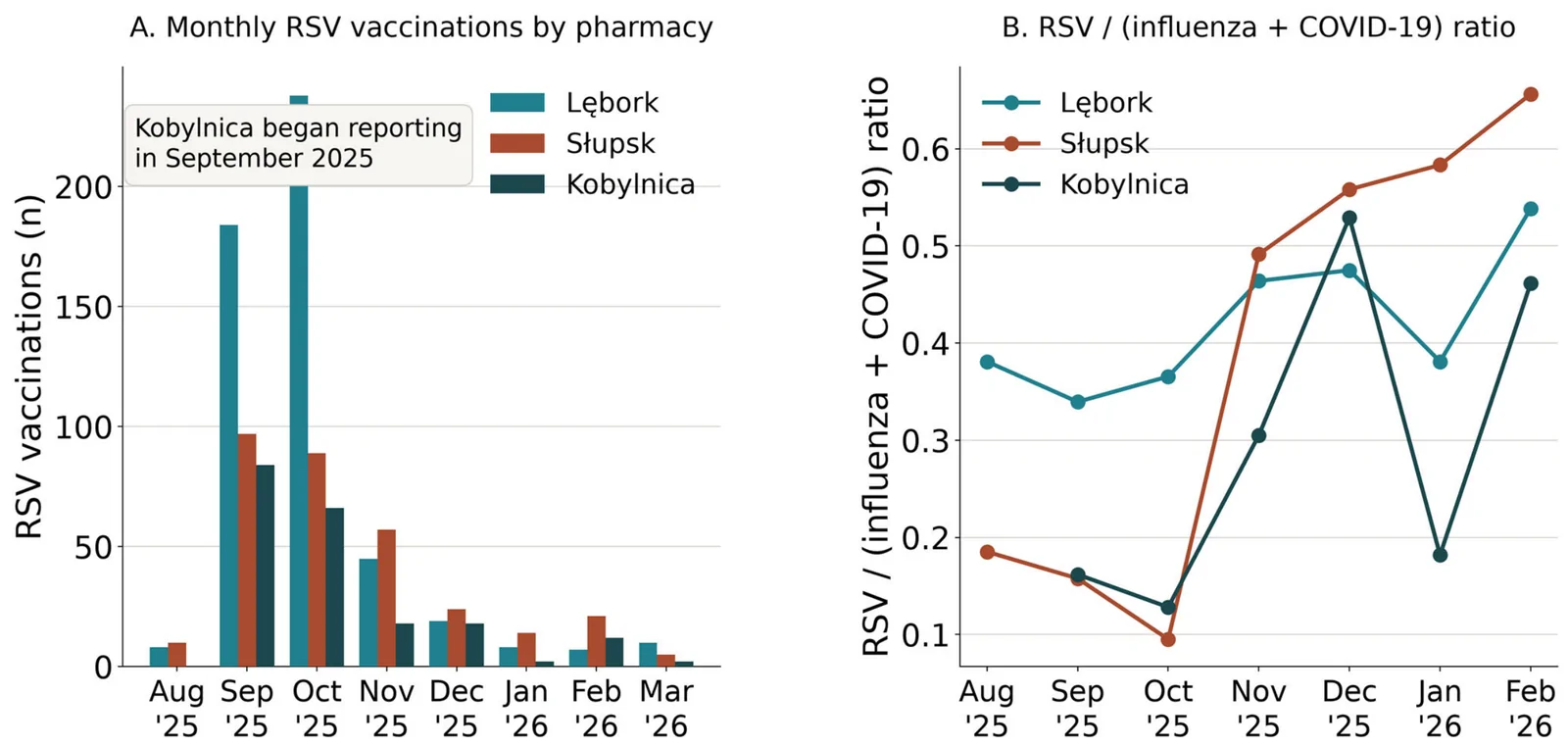
(**A**) Monthly number of RSV vaccinations by pharmacy. (**B**) RSV/(influenza + COVID-19) ratio by pharmacy. Panel (**B**) excludes March 2026, in which influenza + COVID-19 vaccinations fell to 1, 1 and 3 in Lębork, Słupsk and Kobylnica, producing unstable ratios (reported in Supplementary Table S3). Kobylnica began reporting in September 2025; no August 2025 bar is shown for this site.

The exploratory temporal-interaction analysis (Lębork × calendar month) did not show a statistically significant differential trend when all months were included (interaction IRR 0.84; 95% CI 0.69–1.02; *p* = 0.085). However, a sensitivity analysis excluding the unstable March 2026 month indicated that the intervention pharmacy’s relative RSV-rate advantage narrowed over the observation period (interaction IRR 0.79; 95% CI 0.70–0.89; *p* < 0.001), consistent with increasing RSV uptake in comparator pharmacies later in the season. The temporal pattern is shown in Figure 2.

## 4. Discussion

This real-world study found that a structured pharmacist-led recommendation and coadministration protocol was associated with substantially higher use of vaccination opportunities in community pharmacy practice. Compared with routine practice, the intervention pharmacy achieved a higher mean number of vaccine types per patient, a markedly greater proportion of patients receiving multiple vaccines, more frequent same-day coadministration, and a higher RSV vaccination rate relative to respiratory vaccination encounters. Triple-vaccine coadministration visits were observed exclusively in the intervention pharmacy. As the dataset was restricted to patients who received at least one vaccination, these comparisons describe relative differences in vaccination patterns among presenting, already-vaccinated patients across pharmacies, and should not be interpreted as differences in uptake relative to each pharmacy’s total eligible or registered patient population, for which denominators were not available.

These findings support the growing evidence that vaccine uptake depends not only on vaccine availability and reimbursement but also on how systematically eligible patients are identified and offered vaccination [16,24,25,36,37]. Previous systematic reviews have shown that pharmacist involvement increases adult vaccination rates and improves access to immunization services, while implementation studies highlight the importance of workflow design and proactive patient engagement in reducing missed vaccination opportunities [4,5,7,13,14,15]. Our results extend this evidence by suggesting that the organization of the vaccination process itself may influence how effectively community pharmacies convert vaccination encounters into broader protection against multiple vaccine-preventable diseases.

A notable finding was the substantially greater uptake of multiple vaccines in the intervention pharmacy. Nearly three-quarters of vaccinated patients received at least two vaccine types, compared with approximately 43% in the comparator pharmacies, and this association persisted after adjustment for age and sex (Section 3). Similarly, same-day coadministration was recorded for more than 90% of multi-vaccine patients in the intervention pharmacy. These findings are consistent with the concept that every vaccination encounter should be treated as an opportunity to assess eligibility for all recommended vaccines rather than focusing solely on the vaccine initially requested by the patient [24,36,37]. This approach aligns with evidence supporting coadministration as an effective strategy for reducing missed opportunities and improving vaccine coverage in adults [19,20,21,22,23,36].

Several mechanisms may explain the observed differences. The intervention protocol incorporated proactive identification of potentially eligible patients during routine pharmacy interactions, structured review of vaccination history, presumptive recommendation, and a same-day coadministration offer whenever appropriate [24,36,37]. Behavioral research suggests that presumptive recommendations are associated with higher vaccine acceptance than participatory or optional approaches [38]. By combining this communication strategy with active patient identification and a standardized workflow, the protocol operationalized recommendations from implementation science into routine pharmacy practice [13,14,24,36,37]. These findings are consistent with our recent systematic review of pharmacist-delivered vaccination recommendations for herpes zoster, which found that proactive, pharmacist-initiated recommendation strategies may improve confirmed vaccination uptake, while also highlighting the predominance of non-randomized study designs, a substantial risk of bias across most available studies, and the difficulty of isolating the independent effect of pharmacist recommendation within the multicomponent interventions in which it is typically embedded—a limitation directly analogous to the one affecting the present study (Section 4) [39].

The safety of same-day coadministration involving the vaccine types analyzed here is supported by a growing evidence base spanning both younger (18–64 years) and older (≥65 years) adults. For influenza + COVID-19 coadministration, randomized and real-world studies in adults, including the UK ComFluCOV trial [23] and a high-dose quadrivalent-influenza/mRNA-1273 study in adults ≥ 65 [21], have not identified safety signals beyond expected mild, transient local and systemic reactogenicity. For RSV-containing combinations, a phase 3 randomized, placebo-controlled trial of an mRNA-based RSV vaccine coadministered with an influenza or a COVID-19 vaccine in adults ≥50 years found comparable safety and immunogenicity to separate administration [40], and a conference-reported study of a bivalent RSV prefusion-F vaccine coadministered with COVID-19 and quadrivalent influenza vaccines in adults ≥65 years found an acceptable reactogenicity profile [41]. A real-world VAERS-based analysis of triple RSV + COVID-19 + influenza coadministration has similarly reported a favorable safety profile, although this evidence remains hypothesis-generating [42]. Current U.S. CDC clinical guidance explicitly supports coadministration of RSV vaccines with influenza and COVID-19 vaccines in older adults without a required interval [20]. UK national guidance similarly supports RSV–COVID-19 coadministration without a minimum interval, although it advises against routinely scheduling RSV and influenza vaccines at the same visit as a general precaution, while confirming that no specific interval is required should same-day coadministration be clinically preferred (e.g., when a patient is unlikely to return) [43]. A broader safety review of adult vaccine coadministration across multiple combinations similarly reported no consistent excess of serious adverse events [44]. In a related but non-adult population, a systematic review in individuals under 18 years of age found a modest increase in expected, transient reactogenicity when three or more injectable vaccines were coadministered in the same session, but no significant increase in serious adverse events; while this evidence cannot be extrapolated directly to adults, it provides indirect supportive context for multi-vaccine coadministration more broadly [45]. For herpes zoster + influenza coadministration, a large VAERS-based analysis (2018–2024) did not identify a disproportionate safety signal [46]. We note, for balance, that not all guidance is uniform: one provincial immunization advisory committee in Canada has recommended a precautionary interval, rather than routine same-day coadministration, for one specific RSV vaccine product used in long-term-care residents, pending further data, although this recommendation may be shortened in specific situations where the benefit of immediate protection outweighs the risk of delay [47]. More broadly, spontaneous reporting systems such as VAERS [46] are subject to substantial underreporting and reporting bias and cannot, on their own, establish the true incidence of adverse events among vaccinated individuals or a causal relationship between vaccination (including coadministration) and any reported outcome; fatal outcomes reported in elderly vaccine recipients in particular must be interpreted against a high age-related background mortality that is independent of vaccination. Taken together, the available evidence supports the safety of the coadministration patterns observed in our data across both age strata, while underscoring that formal age- and product-specific safety data for some combinations, particularly triple coadministration, remain limited.

The higher RSV vaccination rate observed in the intervention pharmacy is particularly noteworthy. RSV vaccination is relatively new in adult immunization programs and may be less familiar to patients than influenza or COVID-19 vaccination [18,20]. Consequently, uptake may be especially sensitive to healthcare professional recommendation [24,25,37,38]. The finding that RSV vaccination was more frequently delivered alongside other respiratory vaccines suggests that structured recommendation and coadministration strategies may help integrate newly introduced vaccines into routine adult vaccination programs [19,20,24,36,37].

The exclusive occurrence of triple-vaccine coadministration visits in the intervention pharmacy further supports this interpretation. Although the overall number of such visits was limited, their presence only in the protocol-based setting suggests that structured workflows may facilitate delivery of a complete respiratory vaccination package during a single encounter [19,20,24,36,37]. This observation may be relevant for future vaccination programs seeking to optimize protection against multiple respiratory pathogens while minimizing the need for repeated healthcare visits [19,20,24].

At the same time, the low uptake of pneumococcal (Prevenar 13, Pfizer Europe MA EEIG, Brussels, Belgium) and herpes zoster (Shingrix, GlaxoSmithKline Biologicals S.A., Rixensart, Belgium) vaccines across all study sites highlights a structural barrier that is unlikely to be resolved by workflow improvements alone [16,17]. Unlike the COVID-19, influenza and RSV vaccines analyzed here, reimbursement eligibility for these two vaccines in Poland depends on documented medical indications (e.g., specific chronic-disease or immunocompromising conditions) rather than age alone (Section 2.2). Because community pharmacists in Poland do not have routine access to complete medical records, they cannot independently verify these indications and can generally only administer these two vaccines when the patient already holds a physician-issued prescription, in contrast to the proactive, pharmacist-initiated recommendation pathway available for the age-based vaccines [29,30,35]. This limitation is therefore structural rather than a gap in the routinely collected data, and it points to the need for improved pharmacist access to reimbursement-relevant clinical information, or to pharmacy-oriented decision-support and referral tools, if pharmacist-led recommendation strategies are to meaningfully extend to indication-based vaccines. Similar recommendations have been highlighted in pharmacy vaccination guidance documents and policy reports [35,37].

The findings have practical implications for pharmacy-based vaccination services. Importantly, the intervention did not rely on additional staff, new reimbursement mechanisms or expanded legal authority. Instead, it involved reorganization of existing workflows and systematic engagement of the whole pharmacy team [13,14,24,37]. As community pharmacies play an increasingly important role in adult vaccination programs [4,5,13,14,35,37], structured recommendation and coadministration protocols may represent a scalable approach to improving vaccination uptake and reducing missed opportunities in routine practice [24,37].

Several limitations should be considered. First, the observational, non-randomized design precludes causal inference. Second, only three pharmacies were included and the intervention pharmacy was selected pragmatically rather than randomly. It was judged a priori, by the owner and vaccinating pharmacist, to have the least favourable conditions among the three sites for spontaneous vaccination demand (Section 2.1); this qualitative judgement cannot be independently verified, and pre-implementation vaccination data comparable to the study dataset were not available to confirm it—in part because the legal and reimbursement framework governing pharmacy-based vaccination in Poland changed substantially during 2025, making earlier counts, where they exist, not directly comparable to study-period data. As a result, the observed effect cannot be fully separated from site-specific organizational characteristics, local patient-population differences (e.g., age structure, chronic-disease prevalence, socioeconomic factors), or from unmeasured differences in baseline vaccination activity that may have existed before the study period. Third, the intervention was partially confounded by the characteristics of the individual vaccinating pharmacist (e.g., experience, communication style, personal motivation to recommend vaccination) and by local patient-population characteristics that may differ across the three towns, none of which could be measured or adjusted for with the available data. Because only one pharmacy implemented the protocol, these site-specific factors are structurally inseparable from the effect of the protocol itself, and the results should be interpreted as reflecting the combined effect of the protocol and the intervention site’s characteristics rather than the protocol in isolation; this same difficulty in isolating the independent effect of pharmacist-delivered recommendation from co-occurring intervention components has been identified as a pervasive limitation across the broader pharmacist-vaccination literature, not one specific to the present study [39]. Fourth, individual-level clinical information was unavailable, preventing adjustment for comorbidities, risk status and other potential confounders beyond age and sex. Fifth, protocol adherence was not formally assessed at the intervention pharmacy: we do not know whether all seven steps (patient identification, eligibility assessment, vaccination-history review, strong recommendation, coadministration offer, administration and documentation) were consistently delivered, whether adherence changed over the observation period, or which individual components contributed most to the observed effect; prospective fidelity monitoring (e.g., structured checklists or direct observation) is needed in future implementation studies to address this. Sixth, patient identifiers were anonymized separately within each pharmacy and could not be linked across sites. A patient who used more than one of the three study pharmacies during the observation period would therefore be represented as separate, apparently unrelated patients in each pharmacy’s data, which would inflate the total patient count and could artificially reduce the apparent proportion of multi-vaccine or same-day-coadministration patients at any single site (because some of that patient’s vaccinations may have occurred at a different pharmacy); the direction and magnitude of this potential misclassification cannot be quantified with the available anonymized data. Seventh, because the dataset comprised only patients who received at least one vaccination, none of the reported proportions represent uptake relative to each pharmacy’s total eligible or registered patient population, for which denominators were not available; the findings should be read as describing relative differences among presenting, vaccinated patients rather than population-level uptake. Eighth, the routinely collected dataset did not include post-vaccination adverse-event data; therefore, the present study was not designed to assess the safety of vaccine coadministration, and the supportive safety literature discussed above should not be taken to imply that this dataset itself provides safety evidence for the observed coadministration patterns. Finally, inferential analyses were based on a limited number of pharmacy-month observations (n = 23), and despite the use of robust standard errors, the modelling results should be considered exploratory and hypothesis-generating rather than precise effect estimates.

Overall, the consistency of descriptive and model-based findings, including the age- and sex-adjusted analysis of multi-vaccine uptake, supports the interpretation that a structured recommendation and coadministration workflow was associated with more efficient use of vaccination opportunities in community pharmacy practice. Future multicenter studies involving larger numbers of pharmacies, documented pre-implementation baseline data, and prospective assessment of implementation fidelity are needed to determine which protocol components contribute most strongly to improved vaccination uptake.

## 5. Conclusions

A structured, multicomponent pharmacist-led recommendation and coadministration protocol—combining proactive identification of eligible patients, presumptive recommendation and team-based delivery—was associated with higher RSV vaccination rates among vaccinated patients, more multi-vaccine patients and more frequent same-day coadministration than routine practice among patients presenting for vaccination at the study pharmacies. As discussed above, these findings describe relative patterns among vaccinated patients rather than uptake against the total eligible population, and the single-intervention-site design means the protocol’s effect cannot be fully separated from site-specific characteristics. The findings support further evaluation of team-based, proactive-identification workflows in larger multicentre studies—ideally with documented baseline data, randomized or stepped-wedge allocation of pharmacies, and prospective fidelity assessment—together with improved access to reimbursement-relevant clinical information, to inform future efforts to optimise adult vaccination delivery in community pharmacies.

## Figures and Tables

**Table 1:** Baseline characteristics of vaccinated patients.

Characteristic	Lębork (*n* = 931)	Słupsk (*n* = 1424)	Kobylnica (*n* = 893)	Total (*n* = 3248)
Age, years—mean (SD)	65.7 (13.6)	68.9 (12.4)	65.5 (13.3)	67.0 (13.1)
Age, years—median (IQR)	69.0 (60.5–74.0)	71.0 (65.0–77.0)	69.0 (60.0–74.0)	70.0 (62.0–75.0)
Age category, 18–49 years, n (%)	127 (13.6)	119 (8.4)	114 (12.8)	360 (11.1)
Age category, 50–59 years, n (%)	86 (9.2)	126 (8.8)	106 (11.9)	318 (9.8)
Age category, 60–64 years, n (%)	90 (9.7)	102 (7.2)	96 (10.8)	288 (8.9)
Age category, 65–74 years, n (%)	404 (43.4)	592 (41.6)	368 (41.2)	1364 (42.0)
Age category, ≥75 years, n (%)	224 (24.1)	485 (34.1)	209 (23.4)	918 (28.3)
Sex—male, n (%)	443 (47.6)	708 (49.7)	458 (51.3)	1609 (49.5)
Sex—female, n (%)	488 (52.4)	716 (50.3)	435 (48.7)	1639 (50.5)

**Table 2:** Descriptive vaccination activity and coadministration across the three pharmacies.

Metric	Lębork	Słupsk	Kobylnica
Patients with ≥1 of 5 vaccines, n	931	1424	893
Unique patient–vaccine events (5 vaccines), n	1935	2185	1385
Mean number of vaccine types per patient, n	2.08	1.53	1.55
Patients with ≥2 of the 5 vaccines, n	685	606	384
Patients with only 1 of the 5 vaccines, n	246	818	509
Multi-vaccine patients with same-day coadministration, n	635	407	317
Multi-vaccine patients: only separate-day doses, n	50	199	67
Coadministration rate among multi-vaccine patients, %	92.7%	67.2%	82.6%

**Table 3:** Same-day vaccine coadministration combinations by pharmacy (visit-level counts).

Combination	Lębork	Słupsk	Kobylnica	Total
COVID-19 + Influenza	171	215	180	566
COVID-19 + Influenza + RSV	201	0	0	201
Influenza + RSV	140	152	100	392
COVID-19 + RSV	119	31	32	182
Pneumococcal + RSV	2	5	1	8
COVID-19 + Herpes zoster	1	1	2	4
Herpes zoster + RSV	2	3	0	5
Influenza + Pneumococcal	1	2	2	5
Herpes zoster + Influenza	0	1	2	3
COVID-19 + Pneumococcal	2	1	0	3
Herpes zoster + Pneumococcal	1	0	0	1
COVID-19 + Influenza + Pneumococcal	1	0	0	1

Note: Triple-vaccine and COVID-19 + influenza + pneumococcal combinations occurred exclusively at the intervention pharmacy (Lębork).

## Data Availability

The anonymized data were collected by the participating pharmacies from vaccination records reported through the national system Gabinet.gov.pl (Centrum e-Zdrowie) and are subject to a data-use agreement. Patient-level data are not publicly available due to privacy considerations but may be available from the corresponding author upon reasonable request and subject to the applicable data-use conditions.

## Supplementary Materials

The following supporting information can be downloaded at: https://www.mdpi.com/article/10.3390/vaccines14090823/s1, Supplementary Table S1. Poisson generalised linear model for RSV vaccination. Supplementary Table S2. Exploratory temporal-interaction analysis (Lębork × calendar-month). Supplementary Table S3. Monthly aggregated input data per pharmacy. Supplementary Table S4. STROBE checklist. Supplementary Table S5. Structured pharmacist-led recommendation and coadministration protocol. Supplementary Figure S1. Workflow of the intervention protocol.
