# Supplementary material for: Structured Pharmacist-Led Vaccine Recommendation and Coadministration in Community Pharmacies: Real-World Evidence from Poland
Source: Vaccines (Basel). 2026 Sep 19;14(9):823. doi: 10.3390/vaccines14090823 (PMC13611894; doi:10.3390/vaccines14090823)
Supplement: Supplementary file 1 [file vaccines-14-00823-s001.zip › vaccines-4526834-supplementary.pdf]

Supplementary Materials

The following supplementary materials are available: **Table S1.** Poisson generalised linear model for RSV vaccination (incidence rate ratios with 95% confidence intervals, robust standard errors); **Table S2.** Exploratory temporal-interaction analysis (Lębork × calendar-month); **Table S3.** Monthly aggregated input data per pharmacy; **Table S4.** Completed STROBE checklist for observational studies; **Table S5.** Structured pharmacist-led recommendation and coadministration protocol: steps and team responsibilities; **Figure S1.** Schematic of the structured pharmacist-led recommendation and coadministration protocol.

Supplementary Table S1. Poisson generalised linear model for RSV vaccination.

Dependent variable: monthly number of RSV vaccinations. Offset: log(influenza + COVID-19). Reference: comparator pharmacies (Kobylnica + Słupsk); calendar month: September 2025. Robust (sandwich) standard errors are reported owing to overdispersion (Pearson $\chi^2/df \approx 5.5$; deviance/df ≈ 5.5).

Variable	β (log)	IRR	95% CI	p-value
Intervention (Lębork)	0.77	2.17	1.67–2.84	<0.001
October 2025	−0.14	0.87	0.65–1.17	0.351
November 2025	0.68	1.97	1.22–3.19	0.006
December 2025	0.86	2.36	1.39–4.00	0.001
January 2026	0.64	1.89	0.91–3.92	0.089
February 2026	1.08	2.94	1.91–4.52	<0.001
March 2026	2.86	17.44	7.12–42.73	<0.001

The March 2026 estimate is unstable because the offset (influenza + COVID-19 vaccinations) fell to 1–3 per site; it is retained for completeness but is not displayed in Figure 1.

Supplementary Table S2. Exploratory temporal-interaction analysis (Lębork × calendar month).

Derived from the Poisson model in Table S1 with the addition of an interaction between the intervention indicator (Lębork) and calendar month modelled as a continuous (linear) term, testing for a differential temporal trend in the RSV rate between the intervention and comparator pharmacies. Robust standard errors.

Analysis	β (log)	IRR	95% CI	p-value
Lębork × calendar month (all months)	−0.17	0.84	0.69–1.02	0.085
Lębork × calendar month (excluding March 2026)	−0.24	0.79	0.70–0.89	<0.001

The primary analysis including all months was not statistically significant. A sensitivity analysis excluding March 2026, whose offset (influenza + COVID-19 vaccinations) fell to 1–3 per site, showed that the intervention pharmacy's relative RSV-rate advantage narrowed significantly over the observation period, consistent with increasing RSV uptake in the comparator pharmacies later in the season. No formal pre/post parallel-trends test was performed because the protocol was implemented at the start of the observation window and a distinct, extended pre-period was not available.

Supplementary Table S3. Monthly aggregated input data per pharmacy.

Pharmacy	Month	RSV	Influenza	COVID-19	Influenza + COVID-19	RSV rate
Kobylnica	2025-09	84	245	274	519	0.162
Kobylnica	2025-10	66	195	320	515	0.128
Kobylnica	2025-11	18	35	24	59	0.305
Kobylnica	2025-12	18	24	10	34	0.529
Kobylnica	2026-01	2	11	0	11	0.182
Kobylnica	2026-02	12	26	0	26	0.462
Kobylnica	2026-03	2	3	0	3	0.667
Lębork	2025-08	8	21	0	21	0.381
Lębork	2025-09	184	257	285	542	0.339
Lębork	2025-10	238	271	380	651	0.366
Lębork	2025-11	45	48	49	97	0.464
Lębork	2025-12	19	25	15	40	0.475
Lębork	2026-01	8	13	8	21	0.381
Lębork	2026-02	7	11	2	13	0.538
Lębork	2026-03	10	1	0	1	10.000
Słupsk	2025-08	10	54	0	54	0.185
Słupsk	2025-09	97	419	196	615	0.158

Pharmacy	Month	RSV	Influenza	COVID-19	Influenza + COVID-19	RSV rate
Słupsk	2025-10	89	346	590	936	0.095
Słupsk	2025-11	57	72	44	116	0.491
Słupsk	2025-12	24	23	20	43	0.558
Słupsk	2026-01	14	16	8	24	0.583
Słupsk	2026-02	21	29	3	32	0.656
Słupsk	2026-03	5	0	1	1	5.000

Note: Kobylnica's routine reporting in the national system began in September 2025; no August 2025 row is shown for this pharmacy (consistent with Figure 2 and Section 2.1 of the main text). Counts represent monthly vaccine administrations (doses); a patient who received two doses of the same vaccine type within the study period (e.g., the two-dose herpes zoster series) contributes an additional dose to the relevant monthly count. Study-period RSV totals derived from this table therefore correspond to 519, 317 and 202 RSV administrations in Lębork, Słupsk and Kobylnica respectively, which are consistent with, but not identical to, the 519, 317 and 201 unique RSV-vaccinated patients reported in the main text (one Kobylnica patient received two RSV doses during the study period).

Supplementary Table S4. STROBE checklist for observational studies.

Item	Recommendation	Reported in
1. Title and abstract	Indicate study design in title; structured abstract	Title; Abstract
2. Background/rationale	Explain background and rationale	Introduction, paragraph 1–2
3. Objectives	State specific objectives/hypotheses	Introduction, final paragraph
4. Study design	Present key elements of design	Methods 2.1
5. Setting	Describe setting, locations, relevant dates	Methods 2.1
6. Participants	Eligibility, sources, methods of selection	Methods 2.2
7. Variables	Define outcomes, exposures, predictors, confounders	Methods 2.3–2.4
8. Data sources/measurement	For each variable, give sources of data and details of assessment	Methods 2.2

Item	Recommendation	Reported in
9. Bias	Describe efforts to address sources of bias	Methods 2.3; Discussion (limitations)
10. Study size	Explain how study size was arrived at	Methods 2.5
11. Quantitative variables	Explain how variables were handled	Methods 2.4
12. Statistical methods	Describe all statistical methods, including confounding	Methods 2.5
13. Results — participants	Numbers at each stage, flow	Results; Table 1; Table 1 (continued); Table 2
14. Descriptive data	Characteristics of participants and information on exposures	Results; Table 1; Table 1 (continued)
15. Outcome data	Report numbers of outcome events or summary measures	Results; Table 2; Figures 1–2
16. Main results	Give unadjusted and adjusted estimates and CI	Results (age- and sex-adjusted logistic regression); Supplementary Table S1 (Poisson model)
17. Other analyses	Report other analyses done	Results; Table 3 (same-day combination breakdown); Supplementary Table S2 (temporal-interaction)
18. Key results	Summarise key results	Discussion, paragraph 1; Conclusions
19. Limitations	Discuss limitations and potential biases	Discussion (limitations)
20. Interpretation	Give a cautious overall interpretation	Discussion; Conclusions
21. Generalisability	Discuss external validity	Discussion (limitations)
22. Funding	Give funding sources and role of funders	Funding section

Supplementary Table S5. Structured pharmacist-led recommendation and coadministration protocol: intended workflow and team responsibilities (intervention pharmacy, Lębork).

A. Protocol steps

Step	Component	Description
1	Patient identification	During routine pharmacy encounters (including visits for prescription dispensing or medication collection), pharmacy staff were to screen adults for potential vaccination eligibility based on age and other visible criteria, rather than waiting for patient-initiated vaccination requests.
2	Eligibility assessment	The pharmacist assessed eligibility for each vaccine (COVID-19, influenza, RSV, herpes zoster, pneumococcal) based on age, planned and prior vaccinations, reimbursement status and contraindications.
3	Vaccination history review	The pharmacist reviewed recorded status for COVID-19, influenza, RSV, pneumococcal and herpes zoster vaccination to identify vaccines that were due.
4	Strong (presumptive) recommendation	Eligible patients were to receive a clear, proactive recommendation for all due vaccines (e.g., "Based on your age and eligibility, I recommend RSV vaccination together with your influenza vaccination today"), rather than an optional or passive offer.
5	Offer of same-day coadministration	In the absence of contraindications, due vaccines were offered for administration during the same visit; where same-day vaccination was declined or not clinically possible, a subsequent appointment was scheduled and documented.
6	Administration	Eligible due vaccines were administered during the same visit when clinically appropriate and accepted by the patient.
7	Documentation and follow-up	All vaccinations were recorded in the national reporting system (Gabinet.gov.pl, Centrum e-Zdrowie); the patient received aftercare advice and the next recommended doses were planned where applicable.

B. Team responsibilities

Team member	Responsibility
Pharmacy technician / support staff	To proactively identify potentially eligible patients during routine encounters and refer them to the pharmacist.
Pharmacist	Eligibility assessment, vaccination history review, strong recommendation, clinical qualification and vaccination.
Reception / pharmacy staff	Appointment scheduling for subsequent doses.

Note: The protocol was implemented as routine workflow; adherence to the individual protocol steps was not formally measured.

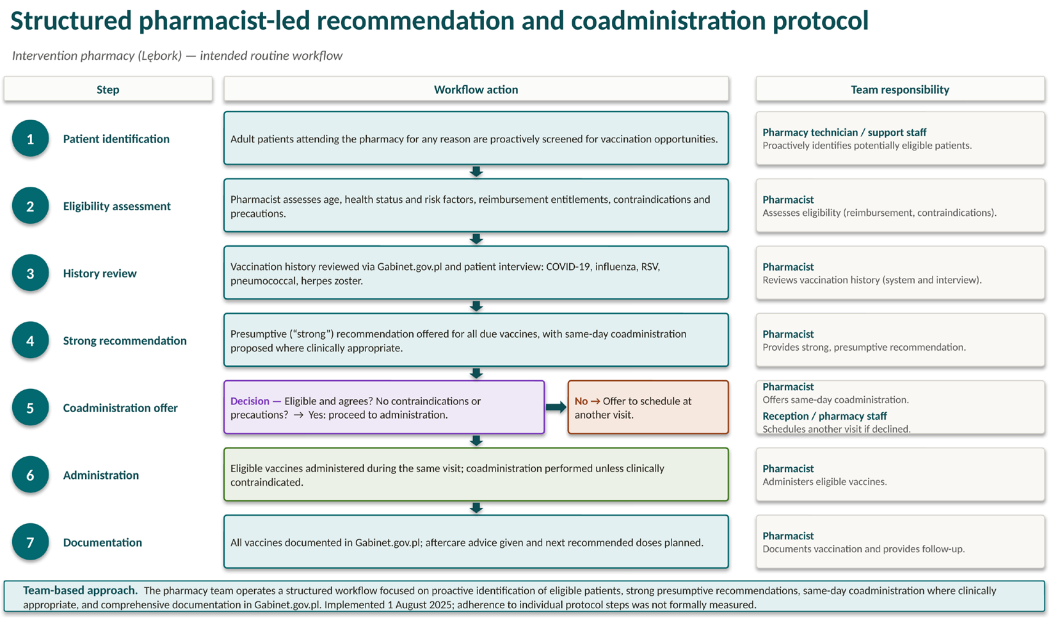

Figure S1. Schematic of the structured pharmacist-led recommendation and coadministration protocol (intervention pharmacy, Lębork). The seven-step workflow comprises (1) proactive patient identification during routine pharmacy encounters, (2) eligibility assessment, (3) vaccination history review via Gabinet.gov.pl, (4) a presumptive ("strong") recommendation for all due vaccines, (5) the offer of same-day coadministration (decision point: eligible and agrees? — yes: proceed to administration; no: offer to schedule at another visit), (6) administration of all eligible vaccines during the same visit, and (7) documentation in Gabinet.gov.pl with aftercare advice and planning of subsequent doses. Team responsibilities are shown in the right-hand column; the protocol was implemented as routine workflow from 1 August 2025, and adherence to the individual protocol steps was not formally measured.
